# Impacts of a Scattered-Site Managed Alcohol Program for People With Alcohol Use Disorder in Halifax, Nova Scotia: A Mixed-Methods Study

**DOI:** 10.1177/29767342261444527

**Published:** 2026-05-26

**Authors:** Candis Lepage, Leah Genge, Thomas D. Brothers

**Affiliations:** 1 Dalhousie University – Department of Emergency Medicine, Halifax, NS, Canada; 2 Dalhousie University – Department of Family Medicine, Halifax, NS, Canada; 3 North End Community Health Centre, Halifax, NS, Canada; 4 Dalhousie University & Nova Scotia Health – Division of General Internal Medicine, Department of Medicine, Halifax, NS, Canada

**Keywords:** alcohol use disorder, harm reduction, program evaluation

## Abstract

**Background:**

Managed alcohol programs (MAPs) are harm reduction services that provide regular, measured doses of beverage-grade alcohol alongside social and health services, aiming to reduce nonbeverage alcohol consumption and harms associated with excessive intoxication and withdrawal. Most MAPs are paired with supportive housing, which can be at a single building (“single-site”) or more uncommonly across multiple apartments (“scattered-site”). The North End Community Health Centre – Mobile Outreach Street Health (MOSH) in Halifax, Nova Scotia, established Atlantic Canada’s first MAP, using a scattered-site model, in April 2020 – during the COVID-19 pandemic. We aimed to describe program participants, summarize outcomes, and understand stakeholder perceptions of program successes and challenges.

**Methods:**

Parallel, mixed methods program evaluation. Quantitative arm involved retrospective review of weekly program assessments documenting presence or absence of outcomes (April 2020-December 2022). Qualitative arm included 4 focus groups with key stakeholders (May-June 2021).

**Results:**

Over the first 33 months, 38 people enrolled in MAP. At the time of our study, 9 had died and 9 left the program. Of the 20 remaining participants, 13 consented to chart review (92% male; age mean 46, SD 11 years) and 9 had weekly outcome data available. Comparing year 1 to year 2, participants had mean person-week prevalence of any nonbeverage alcohol consumption (eg, hand sanitizer): 9% and 3%; acute alcohol-related harms: 25% and 9%; and seizures: 4% and 1%. Mean person-week prevalence of out-of-program drinking was 34% in year 1 and 33% in year 2. Focus groups highlighted MAP improved participants’ health and decreased frequency of nonbeverage alcohol consumption, survival behaviors, and violence. Challenges included out-of-program drinking and lack of social programming.

**Conclusion:**

This scattered-site MAP may support positive health and social outcomes for people with severe alcohol use disorder. Identified program gaps can be explored to better meet participant needs and reduce out-of-program drinking.

**Highlights**

- Quantitative and qualitative data suggest a scattered-site managed alcohol program (MAP) model was successful in supporting individuals with severe alcohol use disorder who have experienced chronic homelessness.
- Long-term participation in MAP was associated with reductions in over-intoxication, nonbeverage alcohol consumption, and acute alcohol-related harms, between participants’ first and second years.
- Stakeholders identified potential limitations regarding eligibility criteria and alcohol choice, and some challenges raised by the scattered-site model including lack of social programming and concerns around managing out-of-program drinking.

## Introduction

Adults experiencing homelessness face disproportionate rates of chronic illness, trauma, mental illness, violence, and have higher rates of substance and alcohol use compared to the general population.^1-4^ Within Canada, between 37% to 73% of adults experiencing homelessness engage in heavy episodic or daily drinking (more than 20 standard drinks per day), interfering with their ability to access and maintain housing.^1-5^ People experiencing homelessness and alcohol use disorder face risks of alcohol-related injuries, exposure to extreme environmental elements, theft, or exploitation;^2,4,5^ people with limited income may need to consume low-cost nonbeverage alcohol (NBA; eg, mouthwash; hand sanitizer) or to engage in theft to obtain alcohol.^4,6,7^ Beyond individual harms, this results in substantial health and social costs, ambulance and emergency department utilization, hospitalizations, and frequent police encounters.^1,3,5,7,8^ Heavy alcohol consumption is also associated with increased rates of mental illness, poor physical health, and chronic diseases,^1,4-6^ as well as alcohol-related harms including withdrawal, seizures, alcohol-related injuries, and alcohol-related mortality.^3-5,7^ As a result, people experiencing homelessness and alcohol use disorders are a priority population for equity-seeking health initiatives.

Managed alcohol programs (MAPs) are harm reduction services for individuals with severe alcohol use disorder, particularly those experiencing homelessness.^2,5,9-13^ MAPs provide participants with regular, measured doses of beverage-grade alcohol at scheduled dispensing intervals to prevent withdrawal, reduce binge drinking episodes and public intoxication, and decrease NBA consumption and alcohol-related harms.^1-3,6,8,10,14^ They often provide housing and primary healthcare services, financial support, cultural programming, social services, and other programming.^2,8^ These programs operate using various models including at residential single-sites, day programs, hospital-based, shelter-based, scattered-site (eg, across multiple housing units), or a mix of multiple settings. MAP operational guidance documents were published for the province of British Columbia in 2020^15^ and nationally in Canada in 2023.^16^ In recent years, there has been a rapid increase in the number of MAPs operating in Canada – many catalyzed by the COVID-19 pandemic. As of 2024, there were 44 MAPs in Canada operating within 8 provinces and 1 territory.^17^ MAPs have expanded across Canada more quickly than other jurisdictions; we are aware of few MAPs in international locations, including 2 in USA; 3 in Australia; and 1 in Spain.^18-20^ Limited uptake internationally may be due to several factors, including the need for further evidence evaluating different MAP models in different settings.

Evidence from a variety of Canadian models suggests MAPs may reduce frequency of binge drinking and NBA consumption, frequency and severity of withdrawal symptoms, and incidence of alcohol-related harms.^5,7,8,10,17,21^ MAP participation is also associated with reduced police encounters.^3,5,7,17,18,21^ Long-term MAP participation (defined by one study as enrollment for more than 2 months), was associated with increased engagement in primary healthcare services.^10^ MAPs are acceptable to participants, with residential-based models being viewed as dignified services that provide safety, increase perceived self-worth, and sense of control.^3,4,9,12,22^ These programs also facilitate opportunities for participants to positively engage with their community, develop therapeutic relationships with healthcare professionals and staff, and build, maintain, and repair family and social relationships.^3,4,7,9,17,21^ Although an emerging evidence-base suggests MAPs are successful in supporting individuals with alcohol use disorder, there is especially limited evidence regarding “scattered-site” models of MAP, with outreach delivery of beverage-grade alcohol to multiple apartment or housing units (often with only 1-2 deliveries per day). We are aware of only 4 other MAPs (located within British Columbia and Saskatchewan, Canada) that included scattered-site deliveries, but no published evaluations of scattered-site models.^11,13,17^ A recent scoping review specifically highlighted the need for more research on scattered-site MAP models.^11^

In April 2020, the Mobile Outreach Street Health (MOSH) program in Halifax, Nova Scotia, Canada established a mixed model (scattered- and single-site) MAP on an emergency basis to support people with severe alcohol use disorder experiencing homelessness. This included providing MAP at both a supportive housing residential site and a scattered-site model delivering alcohol to participants’ independent residences and COVID isolation shelters in the community. This was established urgently during the first wave of the COVID-19 pandemic, in order to facilitate self-isolation and/or quarantine.^18,22^ It is among Canada’s first mixed-scattered site MAP models, and, at the time, there was no national guidance for scattered-site programs. It has since expanded beyond its initial scope to continue to be able to support people experiencing homelessness and severe alcohol use disorder in Halifax. This evaluation serves to contribute to the emerging evidence-base regarding MAPs, address knowledge gaps surrounding scattered-site MAP delivery, and help inform future implementation of MAP models in other jurisdictions.

In this study, we aimed to describe MAP participants; summarize program outcomes (ie, out-of-program drinking, NBA consumption, and acute-alcohol related harms including blackouts, falls, physical altercations, seizures, and withdrawal symptoms); and understand stakeholder perceptions of the MAP’s successes and challenges.

## Methods

This study is a parallel, mixed-methods program evaluation. As the MAP emerged in an emergency response, we took a pragmatic, exploratory approach to evaluation while it was implemented. The quantitative arm comprised a retrospective review of programmatic data, while the qualitative arm consisted of focus groups with key stakeholders representing MAP program staff, community organizations, and municipal and provincial governments.

### MOSH Managed Alcohol Program

MOSH, a program of Halifax’s North End Community Health Centre, established Atlantic Canada’s first MAP on an emergency basis in Spring 2020, to support people with severe alcohol use disorder experiencing homelessness to self-isolate and/or quarantine during COVID-19.^22^ The program was initially supported by Nova Scotia’s COVID response funding and today continues to be funded by the Nova Scotia provincial government. The program aimed to reduce health and social harms associated with severe alcohol use disorder and homelessness and has continued beyond the initial emergency COVID response. It operates by delivering a daily supply of beverage-grade alcohol in the form of beer, wine, and/or fortified wine to participants, as well as provides access to occupational therapy, social work, intensive case management, and primary healthcare. The program does not provide housing, but works in close collaboration with local Housing First and supportive housing providers. It is a mixed scattered-site model, delivering alcohol to both a supportive housing residential site and directly to individuals’ places of residence throughout the community. The fixed site is a “wet shelter” men’s residence, run by a local housing organization. In the fixed site, alcohol is dispensed from a central location and there are opportunities for recreation programming. Scattered-site delivery is to participants’ own apartments – often MAP collaborates with housing organizations so that people can start MAP at the time they are placed in housing. The program has capacity to support 20 to 25 participants at a time.

Potential participants were identified via referral by a network of local community organizations including the North End Community Health Centre and MOSH’s Housing First program, and shelter employees throughout the Halifax area. Potentially eligible participants were known to these organizations and were identified to be living with severe alcohol use disorder, experiencing precarious housing or homelessness, and were considered high risk for experiencing adverse effects from over-intoxication, heavy alcohol consumption, or consuming NBA.

Participants were eligible to participate in MAP if they met all eligibility criteria which included: (1) live within Halifax; (2) chronic homelessness (ie, experiencing homelessness for at least 6 months within the past year or having recurrent experiences of homelessness for at least 18 months within the last 3 years)^23^; (3) severe alcohol use disorder; (4) daily alcohol consumption; (5) history of prior, unsuccessful alcohol use disorder treatment attempts (ie, withdrawal management, psychosocial and pharmacotherapy treatments); (6) experienced acute alcohol-related harms (including seizures, blackouts, delirium tremens, or injuries); (7) demonstrate capacity to consent to program participation; and (8) adhere to program requirements such as no off-program drinking, diversion, or risks to safety of staff or others.

### Quantitative Methods

#### Study Design and Setting

We conducted a retrospective chart review of participants enrolled in the MAP between April 1, 2020, to December 31, 2022. Participants provided individual informed consent for data extraction to be included in this analysis.

#### Data Sources

Data were collected from paper-based and electronic medical record documentation at program intake, weekly outreach assessments, and medical progress notes. The MAP shares documentation with MOSH and the North End Community Health Centre’s primary care electronic medical record.

#### Measures

From intake assessments, we extracted self-reported information on sex, medical history, alcohol dependency, preferred alcohol type/quantity, severe alcohol withdrawal symptoms (seizures, delirium tremens) or drinking to blackout in the preceding 6 months, and other regular substance use. Race and Indigenous identity were not captured consistently and were not included in our analysis. Past medical history (including mental illness diagnoses) was self-reported by participants at program intake and additional diagnoses were obtained from the electronic medical record. Three scales were used to assess baseline alcohol dependency and risk of severe alcohol withdrawal: Prediction of Alcohol Withdrawal Severity Scale (PAWSS), Alcohol Use Disorders Identification Test (AUDIT), and Severity of Alcohol Dependence Questionnaire (SADQ).^24,25^

Weekly assessments captured dichotomous (presence/absence of any) program outcome data including out-of-program drinking, NBA consumption, blackouts, falls, physical altercations, seizures, and withdrawal symptoms. Data included a mix of self-report and observations by outreach workers, and referred to a time period of the last 7 days. Out-of-program drinking was captured by asking participants, “have you consumed alcohol outside of your MAP dosage within the last 7 days.” If reported as yes, a second question was asked if the alcohol consumed was beverage or nonbeverage alcohol. These assessments did not include any more detailed information about the type/quantity of beverage alcohol consumed. We developed a combined variable of acute alcohol-related harms for the purpose of this analysis, which consisted of the presence of any of blackouts, falls, or physical altercations during that week. In weeks where no outcomes were recorded, the outcome was assumed to be absent. Participants’ alcohol dosages were not reliably recorded in these data sources during this time; we could not include alcohol dosage in this analysis.

#### Analysis

We generated descriptive summary statistics for the sample. We assessed the person-week prevalence of NBA (eg, mouthwash, hand sanitizer) consumption, acute alcohol-related harms, seizures, and out-of-program drinking and calculated the mean person-week prevalence across participants’ first year (ie, 0-52 weeks) and second year (ie, 53-104 weeks) in MAP. Due to the small sample size, in this exploratory evaluation we compared means descriptively without tests of statistical significance. Analyses were conducted using Microsoft Excel – Data Analysis ToolPak ®.

### Qualitative Methods

The first author (C.L.) conducted 4 focus groups via Microsoft Teams with key stakeholders identified by the MAP leadership. Focus groups were held on May 26th, June 3rd, June 10th, and June 14th, 2021. A pragmatic, purposive sampling approach was used; the MAP leadership team (consisting of a program manager, occupational therapist, registered nurse, case manager, and physician) identified and invited key stakeholders who they felt would provide actionable data during the emergency response. Given this, we did not seek thematic saturation within focus groups.

A total of 15 individuals participated in focus groups, representing MAP staff, community partners, and government representatives. MAP staff were identified for their role in scattered-site MAP delivery, which included the MAP program manager, harm reduction outreach coordinator responsible for daily operations, harm reduction outreach workers, case managers, occupational therapist, social worker, registered nurse, and a social work student. Community stakeholders were individuals employed by organizations serving MAP participants including the North End Community Health Center, the Street Navigator Outreach Program, Out of the Cold Community Association, and Shelter Nova Scotia. These individuals held various roles within their organizations including Executive Directors, Case Managers, Social Workers, and Housing Managers. Governmental stakeholders were staff of the Halifax Regional Municipality and the Province of Nova Scotia – Department of Housing.

Focus groups were 90-minutes and used a semi-structured interview guide developed with input from MAP leadership, related to program impact on participants’ health and social outcomes, alcohol consumption, behavior, access to healthcare, as well as challenges or potential gaps related to program delivery. Participants were informed that focus group discussions would be used to inform the MAP evaluation and later compared with quantitative data to assess clinical, social, and health-related outcomes experienced by participants. Focus groups were conducted and analyzed by one researcher (C.L.), independent of the MAP leadership team. Focus groups were audio recorded and transcribed verbatim. C.L. then conducted qualitative description via thematic analysis, by rereading all the transcripts, line-by-line open coding, and organizing codes to identify themes. Emerging qualitative findings were shared with MAP and North End Community Health Centre staff in November 2022 for community input and as member-checking. No major changes were suggested to the thematic analysis findings.

### Reflexivity Statement

The authors are all physicians who value health equity and wish to improve the quality of health services for people experiencing marginalization and exclusion, including people with housing insecurity and alcohol use disorder. C.L. is an emergency medicine resident physician with a special interest in addiction medicine. She led the qualitative component of the study while working as a program evaluator, and the quantitative component during medical school. L.G. is a family physician and addiction medicine specialist, and a physician with MOSH; she helped to found the MAP under evaluation here and is experienced in development and leadership of substance use harm reduction programming. T.D.B. is a general internist and addiction medicine physician-scientist, experienced in substance use health program leadership and evaluation. He worked clinically with MOSH as part of the COVID response and provides general internal medicine consultations to MOSH patients (including MAP participants). We recognize the MAP was implemented as an imperfect emergency response, and the main aims of the program evaluation were to identify opportunities to strengthen the program.

## Results

### MAP Participants

**Figure 1.**
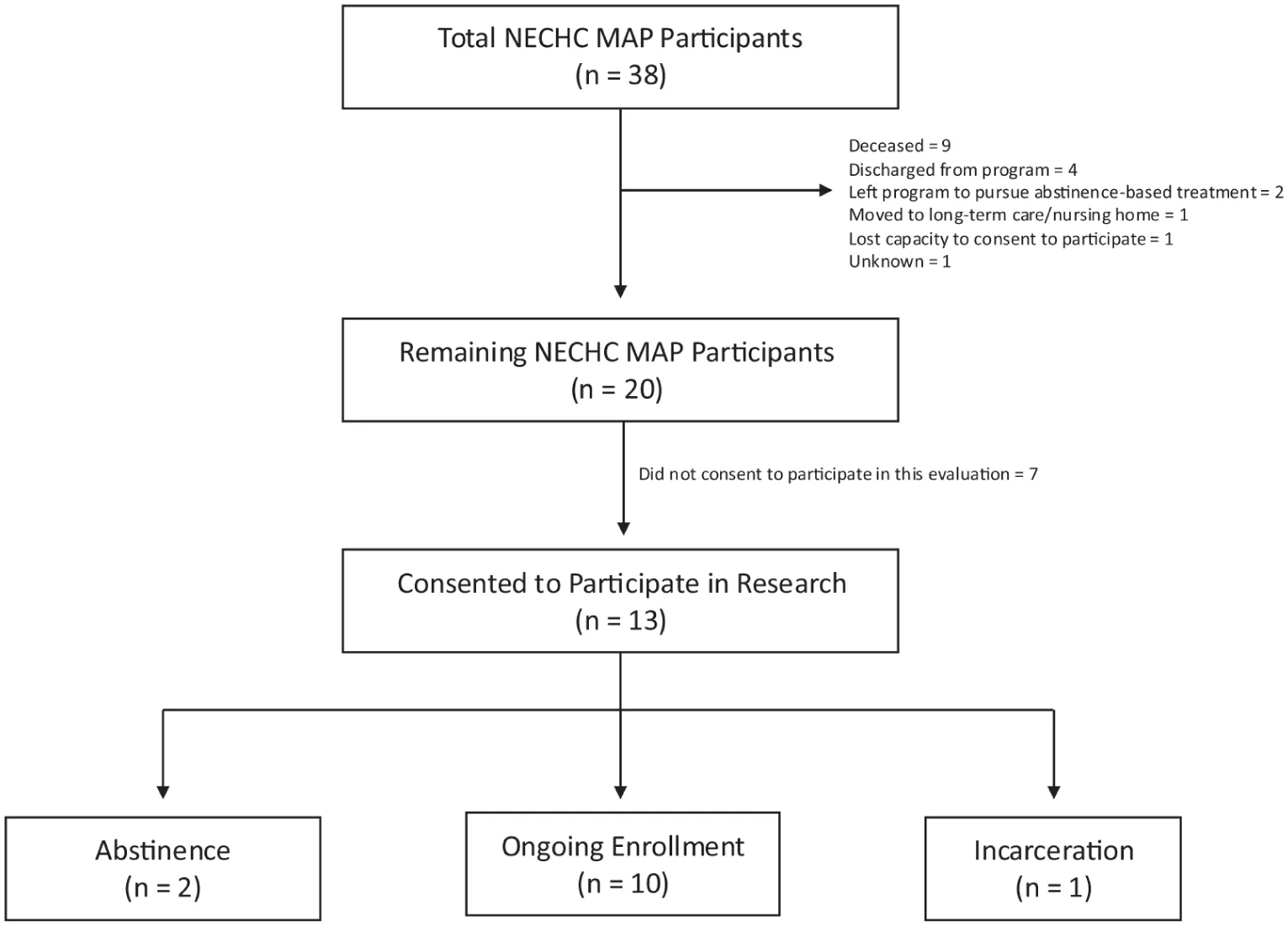
Description of managed alcohol program participant enrollment.

Over the first 33 months of the MAP, 38 individuals enrolled (Figure 1). Nine died and 9 left the program: 1 entered long-term residential care/nursing home, 2 pursued abstinence-based treatment, 1 lost capacity, and 4 were discharged. Reasons for discharge included repeated diversion of alcohol, repeated drinking outside of the program causing bodily harm, and change in drinking behaviors no longer consistent with eligibility criteria (ie, intermittent binge drinking). For one participant, the reason for program cessation was not clearly documented. Of the remaining 20 participants enrolled in MAP, 13 (65%) consented to be included in this study. We did not collect reasons for declined participation for the 7 remaining participants.

Participant health and sociodemographic characteristics at intake are summarized in Table 1. The majority of participants were male (92%; n = 12), mean age 46 (SD 11) years. At intake, 15% (n = 2) of participants had cirrhosis. Many had mental health concerns: 38% (n = 5) with depression and/or anxiety and 23% (n = 3) with posttraumatic stress disorder.

**Table 1.** Baseline Characteristics of Managed Alcohol Program Participants.

Characteristic	n (N)	Mean (n, range)
Age, years	13 (13)	45.9 (32-66)
	n (N)	Frequency (%)
Sex		
Male	12 (13)	92.3
Medical history		
Pancreatitis	1 (13)	7.7
Cirrhosis	2 (13)	15.4
Posttraumatic stress disorder	3 (13)	23.1
Depression	5 (13)	38.5
Anxiety	5 (13)	38.5
Schizophrenia	1 (13)	7.7
Bipolar disorder	1 (13)	7.7

Participant substance use at intake is presented in Table 2. Mean scores for PAWSS was 7.7 (range 4-9), for AUDIT was 38 (range 28-40), and for SADQ was 49.5 (range 30-58). At enrollment, 85% (n = 11) of participants reported beer as their preferred alcohol and 3 (23%) primarily consumed spirits. Nine participants (69%) consumed NBA on a regular basis. In the 6 months prior to MAP enrollment, 92% (n = 12) reported experiencing blackouts and seizures, and 31% (n = 4) reported experiencing delirium tremens. Other substance use was prominent, with 54% (n = 7) of participants using cocaine, crack, and/or cannabis on a regular basis (Table 2).

**Table 2.** Baseline Alcohol and Substance Use of Managed Alcohol Program Participants.

Characteristic	n (N)	Mean (n, range)
Alcohol dependency assessments^a^		
PAWSS score^b^	10 (13)	7.7 (4-9)
AUDIT score^b^	10 (13)	38 (28-40)
SADQ score^b^	10 (13)	49.5 (30-58)
	n (N)	Frequency (%)
Preferred type of alcohol		
Fortified wine	2 (13)	15.4
Spirits	3 (13)	23.1
Beer	11 (13)	84.6
Nonbeverage alcohol	9 (13)	69.2
Alcohol-related symptoms		
Withdrawal seizures	12 (13)	92.3
Blackouts	12 (13)	92.3
Delirium tremens^c^	4 (13)	30.8
Other substance use		
Opioids	2 (13)	15.4
Cannabis	7 (13)	53.8
Cocaine/Crack	7 (13)	53.8
Benzodiazepines	1 (13)	7.7
Hallucinogens	1 (13)	7.7

a Sample size for this calculation was n = 10, 3 participants did not complete all 3 assessments for alcohol dependency at intake. ^b^ PAWSS maximum score = 10; AUDIT maximum score of 40; SADQ maximum score of 60. ^c^ History of delirium tremens were self-reported by participants to MAP frontline staff.

Abbreviations: PAWSS, Prediction of Alcohol Withdrawal Severity Scale; AUDIT, Alcohol Use Disorders Identification Test; SADQ, Severity of Alcohol Dependence Questionnaire; MAP, managed alcohol program.

### Quantitative Findings

**Figure 2.**
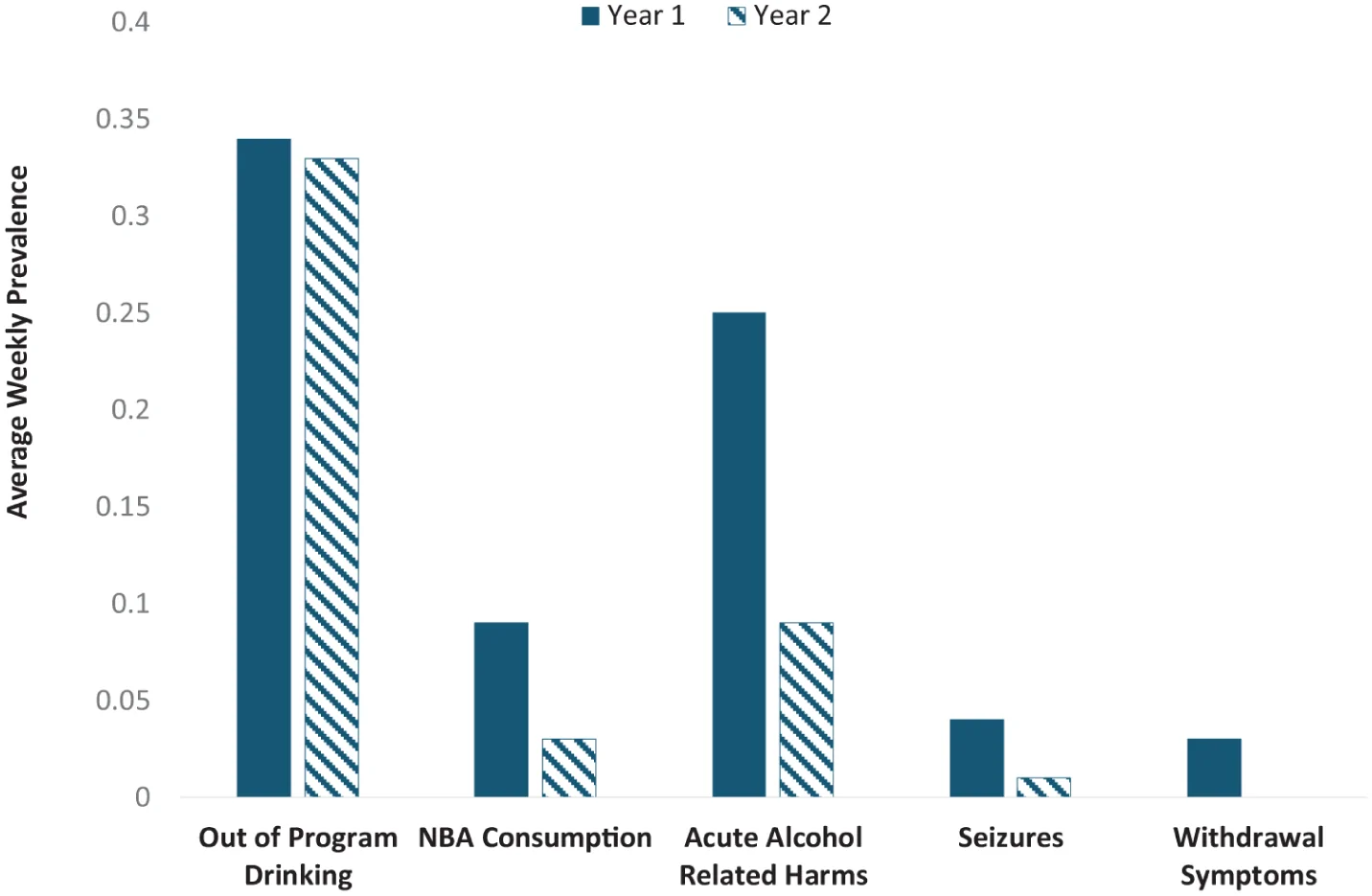
Average weekly prevalence of program outcomes for managed alcohol program participants in year 1 and year 2.

Nine participants had weekly program assessment data available for analysis; the remaining 4 were followed through a different primary care clinic and weekly program outcome data was not retained. At the time of analysis, the 9 participants had been enrolled in MAP for a median of 78 weeks (range 30-104 weeks). Many weeks had missing data (ie, up to 62% of all potential weeks enrolled in MAP). We summarized outcome data only from nonmissing weekly assessments. Comparing participants’ first versus second years in MAP, mean person-week prevalence of any NBA consumption was 9% versus 3%, and any out-of-program drinking was 34% versus 33% (Figure 2). Mean person-week prevalence of acute alcohol-related harms (blackouts, falls, or physical altercations) was 25% in year 1 and 9% in year 2; while seizures were 4% versus 1% and any withdrawal symptoms were 3% versus 0% (Figure 2).

### Qualitative Findings

Focus groups with stakeholders provided insights on early impacts of the MAP. Stakeholders felt participation in MAP positively influenced alcohol consumption patterns, improved participants’ physical and mental health, reduced alcohol-related harms, positively influenced participant behaviors, and helped to foster improved access to primary healthcare. They also raised concerns about restrictive program eligibility (ie, exclusion of individuals living in encampments or with primarily binge drinking patterns), and with the program’s ability to manage over-intoxication or out-of-program drinking, and limited social programming in a scattered-site, outreach model.

#### Alcohol and NBA Consumption Patterns

Stakeholders felt MAP participation reduced instances of over-intoxication and NBA consumption compared to their prior consumption patterns. With reduced over-intoxication, stakeholders perceived participants experienced fewer acute alcohol-related harms including injuries and falls and fewer episodes of withdrawal. A MAP staff member spoke of how the consistent delivery of beverage grade alcohol allowed long-term NBA consumers to shift toward primarily consuming beverage-grade alcohol, reducing the risk of severe intoxication and associated health consequences:

The NBA [non-beverage alcohol] aspect again, due to financial constraints or just their preference over the years from needing to turn to NBA, we [MAP staff] were able to kind of transition a lot of our folks [participants] off of that [non-beverage grade alcohol] – MAP Staff Member

Although the program was viewed as successful in shifting alcohol consumption patterns, some staff found it difficult for some participants to consume only the program-provided beverage alcohol, as options were limited to beer and wine. This was particularly notable for participants with an established preference for spirits, which were not offered within the program at the time:

We had a gentleman who would repeatedly dip [drink out-of-program alcohol] on the program and drink [spirits] every cheque month at the beginning just ‘cause that’s what he drank for so long and the beer wasn’t doing it for him and the wine hurt his stomach – MAP Staff Member

Staff at the MAP supportive housing residential site spoke to difficulties they had with some participants who were unable to properly ration their daily supply of alcohol, at times resulting in the participant consuming additional (“out-of-program”) alcohol and becoming intoxicated. This identified a need to explore additional alcohol offerings and begin divided-dose delivery.

#### Participant Behaviors

Stakeholders felt MAP reduced participants’ exposure to and participation in violence, illegal activity, damage to residential property, and survival behaviors. One supportive housing residential-site staff member spoke about how this supported participants to maintain housing:

Some folks who are at [Building Name], I believe that if they were not on MAP they would lose their residency due to their behaviors when they’re over intoxicated – Supportive Housing Residential Staff Member

Having consistent access to beverage alcohol largely eliminated the need for participants to engage in panning or collecting recyclables or engage in criminalized behaviors (i.e. theft) to meet their daily alcohol needs:

The benefit of folks not having to go out and do those high-risk behaviors to pan or to do whatever they were doing, to get a bottle, that was really helpful for them – Community Stakeholder

At times, not having to dedicate large amounts of time to securing alcohol caused some participants to experience a sense of boredom, felt by stakeholders to be partially attributed to a lack of built-in social programming within the program:

Once our participants become a lot more stable, we have taken away certain things in their days like that would fill their day. Whether it’s like panning, whether it’s out being social, whether it’s hustling, we’ve taken away like a lot of social activity from our participants, and so now to be honest we deal with a bit of like boredom – MAP Staff Member

#### Improved Health and Health Care Access

Stakeholders reported MAP allowed participants to attend medical appointments and improve their health (eg, take regular medications), rather than having to spend time engaging in survival behaviors to acquire alcohol. The provision of onsite, wrap-around supports built into the program also supported engagement in primary healthcare, as well as fostered new linkages to allied health care services including occupational therapy, intensive case management, and physiotherapy:

The tethers that come with the MAP program like OT [occupational therapy] and Nursing, they’ve been able to do on-site visits, that’s been great for folks who had poor attendance at appointments before because they didn’t want to go out or they had to make sure they had alcohol, so they were panning or working or bottling – Community Stakeholder

Stakeholders highlighted that MAP participation also supported improved mental well-being, by reducing anxiety regarding how participants would safely source, store, and consume their daily alcohol:

It’s basically helped to improve their mental health. . .Like I think of a participant who described it as having a significant impact on his anxiety because a lot of his experiences about having to figure out how to get alcohol and then also being able to kind of keep their stash and not worrying about spilling it, or losing it, or having it stolen – Community Stakeholder

#### Restrictive Program Eligibility

Other challenges identified among stakeholders included restrictive program eligibility criteria. Stakeholders felt eligibility criteria should be expanded to include individuals experiencing homelessness (ie, those living in tents or temporary structures), living in shelters, and those who engage primarily in binge (nondaily) drinking; they noted they understood this may require different models of MAP, beyond daily delivery to participants own apartments.

Community stakeholders also expressed frustrations with the current disconnect between provincial long-term care (nursing home) placement policies and MAP delivery. As alcohol use disorder is a lifelong condition for some people, MAP may be a harm reduction intervention participants engage with until their end-of-life. Stakeholders spoke of a participant who required a transition to long-term care, however, was no longer able to access MAP within the facility, thereby forcing them to be discharged from the program in order to secure housing.

## Discussion

We conducted a pragmatic, exploratory mixed-methods evaluation of a mixed scattered-site MAP in Halifax, Canada, that emerged in response to the COVID-19 pandemic. We found that participants in the MAP were almost exclusively male, had high rates of comorbid physical and mental illness, and high severity of alcohol dependence scores. Nearly a quarter (24%) of participants died within the first 33 months of the program, and some participants left the program to pursue abstinence-based treatment or due to cognitive impairment. Mean person-week prevalence of NBA consumption, acute alcohol-related harms, and withdrawal symptoms were nominally lower during MAP participants’ second year in the program compared to their first year. Out-of-program drinking was common, occurring in approximately one-third of nonmissing weekly assessments. Stakeholder perceptions were consistent with these quantitative findings and also identified important limitations to MAP delivery around alcohol choice and programming. Overall, our findings highlight the successes and challenges of a scattered-site MAP in Atlantic Canada and suggest that expansion of scattered-site MAPs in Canada may be a viable approach to help improve program access among rural and under-serviced communities.

Our findings of potential positive health and social outcomes among MAP participants is consistent with prior research, though quantitative conclusions are limited by lack of a control group and much missing data.^8,10,21,22,26^ Most prior quantitative positive findings come from uncontrolled (or before-and-after) observational studies, like our study design here. Qualitative and mixed-methods MAP research has contributed to program theories positing MAP is effective if it can promote autonomy, facilitate hope and purpose, and provide access to health and social services in a manner that is not contingent on sobriety.^4,9,12,27,28^ Our qualitative focus group findings that MAP supports people to maintain their scattered-site housing is also consistent with prior research on a single-site MAP.^29^ We are not aware of prior evaluation data specifically including scattered-site models.

In terms of program reach, our finding that a large majority of participants were male is consistent with other MAPs (eg, 84% in a large sample of MAP participants across Canada).^8,10,30^ Reasons for this gender difference partly reflect higher rates of severe alcohol use disorder and homelessness among men compared to women.^23,31^ Most people being referred to the MOSH MAP for consideration of enrollment are men. The main fixed-site building supported by MAP originated as a men’s-only “wet shelter” and remains a men’s-only building. The MAP has since implemented policies to prioritize referrals for women, nonbinary people, and individuals from equity deserving groups. The only other MAP in Atlantic Canada (in St. John’s, Newfoundland) specifically serves women and gender-diverse individuals only.^32^ The MOSH MAP also served people at high risk of dying; notably, 9 of 38 (24%) participants died within the first 3 years of the program. Prior MAP evaluations have not typically described the proportion of participants with co-occurring physical and mental health conditions, nor the prevalence of cirrhosis.^11^

Although many positive outcomes were identified through this evaluation, delivery of Atlantic Canada’s first scattered-site model was not without some challenges. Out-of-program drinking among MAP participants was common and has been documented in other MAP evaluations. In a post-hoc analysis of their longitudinal, controlled study of MAP participants across Canada, Stockwell et al found greater improvements in health and social outcomes among participants in programs that proactively limited out-of-program drinking.^10^ In response to identifying out-of-program drinking, staff in the MOSH MAP have a discussion with participants to determine if needs can be met in other ways. Often, people are drinking more due to life stressors and staying connected with the program is still beneficial to reduce harm. If out-of-program drinking is causing harm, the MAP follows a “three strikes” policy. After 3 documented instances of out-of-program drinking, participants take a time-limited break from MAP to help understand if MAP is still beneficial to reduce harm. Another challenge occurs when participants develop cognitive impairment and are ineligible to go to nursing homes in Nova Scotia. Long-term care facilities in Nova Scotia currently do not accept anyone with active substance use, including alcohol consumption; this presents a major gap in housing and healthcare for frail elderly people who use alcohol and other drugs. Considering this, it is often deemed safer for these participants to remain in MAP even if they are drinking outside the program – until cognitive impairment becomes even more severe and people may be admitted to hospital and/or eventually placed into a nursing home with an abstinence-based care plan.

While all MAP participants in our study had access to primary care, social work, occupational therapy, and case management, stakeholders highlighted the role of boredom and a need for more robust social programming. A recent report from the Ottawa Inner City Health fixed-site MAP highlights the role of social and life-skills programming in their fixed-site residential buildings, which is more challenging to deliver in a scattered-site model.^33^ The fixed-site supported by the MOSH MAP has recreation and socialization opportunities, including cribbage tournaments and guitar nights, that are not easily accessible to participants in scattered-site apartments. A fixed-site model with central alcohol dispensation may also promote more social interaction than a scattered-site model where alcohol is delivered to people who may otherwise be isolated in their homes.

Other challenges identified among stakeholders included restrictive program eligibility criteria, need for expanded alcohol options, ability to manage complex out-of-program drinking behaviors, and a disconnect between nursing home/long-term care and home care policies and MAP delivery. Stakeholders identified the need for additional alcohol offerings, including spirits, during the time of this study within the program. The MOSH MAP only offered beer and/or wine for a participant’s supply of beverage grade alcohol. The program has since expanded to allow spirits with multiple daily dispenses (3-4 times per day) at the fixed-site building, which may improve program engagement and reduce out-of-program drinking. MAPs in Canada that include spirits typically provide small volumes on an hourly basis, which was not achievable in this setting. The initial exclusion of spirits was intentional in this MAP based on their model of delivery (daily drop offs) and concern for potential over-intoxication; however, this highlights that a range of models may be needed to accommodate participants’ needs, particularly among individuals with heavy spirit or NBA consumption, and/or medical issues that require volume reduction in MAP dosing (eg, hyponatremia).

### Limitations

Our study had several important limitations. First, while we included the perspectives of program staff and community partners, we did not include the perspectives of MAP participants themselves. We prioritized focus groups with stakeholders while the program was being rolled out in an emergency response context, and understanding the perspectives of program participants is an area of planned research. Second, for the quantitative arm of the study we encountered a small sample size (n = 9 with non-missing data), limiting statistical power and generalizability. Third, we extracted data retrospectively from weekly assessments and medical records; these records were kept for programmatic reasons and were not designed for research. Data from weekly assessments relied on self-reported data, carrying an inherent risk for response bias that may include under-reporting of negative outcomes such as experiencing acute alcohol-related harms or failure to recall outcomes within the past 7 days during weekly assessments, potentially impacting our findings. This risk was mitigated were possible with outreach workers having the ability to document in the notes section of weekly assessments any concerns they had related to out of program drinking, emergency service utilization, or signs of over-intoxication based on assessment of the participant during the home visit. We assumed that when an outcome or issue was not recorded in the clinical assessment it did not happen. It is possible that we under-estimated the prevalence of outcomes that were not captured during the weekly assessment. Missing data was also very common, illustrating the competing demands and limited resources while standing up the program during the COVID-19 pandemic. Our summary statistics of mean person-week prevalence presume that the missing assessments were missing completely at random; otherwise, missing data may have led to over- or under-assessment of outcomes. Use of these programmatic outcomes (weekly presence/absence) also limits comparison with prior research on MAPs, which assessed outcomes including number of drinking days, drinks per drinking days, or alcohol-related harms in the prior 30 days.^10,11^ We were also unable to include data on participants’ alcohol dosage. Fourth, in this pragmatic evaluation we were unable to include a control group of similar people who would have been eligible for the MAP but did not enroll (or who were provided MAP without supportive housing). This means that we cannot disentangle potential effects of MAP alone from supportive housing, and that positive outcomes over time may in part reflect regression-to-the-mean rather than direct effects of MAP. Fifth, potential improvements in quantitative outcomes from year 1 to year 2 may reflect an element of survivorship bias, as participants benefiting from the program were more likely to stay enrolled long-term. Together, these limit our ability to attribute causal effects of MAP participation on health and social outcomes in the quantitative analysis.

## Conclusion

The mixed, scattered-site MAP appeared successful in supporting individuals with severe alcohol use disorder experiencing chronic homelessness in Halifax, Nova Scotia, who faced multiple barriers to care. MAP supported participants in achieving positive health and social outcomes. Identified programming gaps can be explored to better meet the needs of some participants and reduce out-of-program drinking and complex behaviors. As the housing crisis continues to impact communities across North America, the demand for MAP will continue to grow. Based on the success of this model, other communities could consider implementing scattered-site MAPs to improve access to care and social supports for people living with severe alcohol use disorder and experiencing housing instability.
